# On the removal efficiency of copper ions in wastewater using calcined waste eggshells as natural adsorbents

**DOI:** 10.1038/s41598-023-27682-5

**Published:** 2023-01-09

**Authors:** Ming-Yu Chou, Tan-Ang Lee, Ying-Shen Lin, Shan-Yin Hsu, Ming-Fu Wang, Po-Hsien Li, Ping-Hsiu Huang, Wen-Chien Lu, Jou-Hsuan Ho

**Affiliations:** 1 International Aging Industry Research & Development Center (AIC), Providence University, Taichung, 43301 Taiwan; 2 Department of Food Science, Tunghai University, Taichung, 407224 Taiwan; 3 Ph.D. Program in Health and Social Welfare for Indigenous Peoples, Providence University, Taichung, 43301 Taiwan; 4 Department of Food and Nutrition, Providence University, Taichung, 43301 Taiwan; 5 School of Food, Jiangsu Food and Pharmaceutical Science College, Huai’an, 223003 Jiangsu Province China; 6 Department of Food and Beverage Management, Chung-Jen Junior College of Nursing, Health Sciences and Management, Chia-Yi City, 60077 Taiwan

**Keywords:** Environmental sciences, Materials science

## Abstract

Eggshells offer many advantages as adsorbents, such as affordability without special preparations other than pulverization and calcination. However, the manufacturing industry generally has a severe problem with high concentrations of heavy metals in wastewater. The purpose of this study was to use eggshell byproducts and calcined eggshell treatment for the adsorption of copper in an aqueous solution. The reaction time, metal concentration, adsorbent dose, temperature, and pH were evaluated using primary factors followed by the response surface method (RSM) to investigate the optimum conditions for eggshell byproducts and calcined eggshell adsorption treatment. The results of the one-factor-at-a-time experiment showed that the optimal adsorption rate was obtained from treatment at 24 h, 25 mg/L, 10 mg, and 25 °C. In addition, the effect of pH on the adsorption rates of eggshells and eggshells with membrane were detected at pH values of 5 and 5.9 and found to be 95.2, 90.5, and 73.3%. The reaction surface experiment showed that the best adsorption rate reached 99.3% after calcination at 900 °C for 2 h and a 20 min reaction. The results showed that eggshells, eggshell membranes, eggshells with membrane, and calcined eggshells could be applied to remove copper ions from industrial wastewater. The adsorption capacity of the calcined eggshell is better than that of the non-calcined eggshell and has good neutrality in acidic industrial wastewater. Therefore, it is convenient and practical for practical production and application. Likewise, this study conveys promising findings in the context of improving wastewater treatment based on a circular economy approach to waste reuse in the food industry and represents a valuable direction for future research.

## Introduction

Eggshells have been considered a waste generated primarily by the poultry and food industries, with a global annual production of approximately 110 billion tons of eggshells reaching landfills^1,2^. However, its composition of 90–98% CaCO_3_, more highly bioavailable calcium than the commercially available limestone, or coral sources, allows for easier absorption^2–7^. Evidence from clinical studies indicates that humans who receive calcium from eggshells have increased bone density, improvements in brittle nails, hair, constipation, and asthma, and even benefit from the removal of radioactive elements^2,8,9^. The various applications of eggshell materials for recycling include wastewater treatment, antibacterial agents, air quality management, biodiesel fuel, electrochemistry, medicine, and food production^3,9–14^. The nanocatalytic system for conversion of biomass into advanced material has proven to be a valuable option (such as calcination and catalysis allow the interaction between both components), yet further efforts are required to improve processes so that their manufacturing costs are well-controlled^9^.

Human-caused heavy metal pollution began in the late eighteenth century and increased rapidly throughout the nineteenth and twentieth centuries. As agricultural and industrial activity expands, the inappropriate discharge of industrial wastewater into the environment has resulted in heavy metals presently being a global issue for the health of humans and other organisms^15–17^.

Activated carbon has been commonly used as an effective adsorbent; unfortunately, it is cost- prohibitive, as it requires a high temperature for carbonization and loss of operation during combustion^7,18^. An adsorption process should feature the most advanced technology in terms of simple design, minimal operational cost-effectiveness, high efficiency, low reagent consumption, and environmental friendliness^6,15,19,20^. Moreover, removing pollutants from water has been an extremely challenging task, and many research endeavors have focused on at least two formulations, each with advantages and disadvantages^21^. In addition, novel studies in the chemical adsorption field have also included (1) the conversion of titanium gypsum (TiG) into an efficient adsorbent for phosphate recovery^22^; (2) phenolic compounds—tannins for precipitation of proteins as sludge conditioning^23^; (3) industrial red mud particle waste used to adsorb Pb^2+^, CD^2+^, and Cu^2+^^24^; (4) C_2_N and g-C_3_N_4_ to separate nitrate and nitrite contaminants from aqueous solutions^25^; (5) ultrasound combined with seed material facilitates phosphorus recovery from swine wastewater by sulforaphane crystallization^26^; (6) use calcined eggshells to remove phosphate, phosphorus, cadmium, and dyes from aqueous solution^14,27–29^. Because of the limited amount of freshwater, the severe effects of industrial pollution, and the increasingly stringent regulations in various countries, water resource management and improvement have become vital to enterprises and scientists^5,21,30^. The current situation is critical for encouraging waste prevention lifestyles at the source and urgently developing technologies that will allow for environmentally and economically sustainable waste disposal^31^.

The adsorbent is a solid substance for removing contaminants from liquids or gases that could be hazardous in the environment. In practice, the adsorption and removal process of the pollutants depends on the characteristics of its physiochemistry substantially^32,33^. Therefore, a commercial adsorbent's outstanding performance properties are high selectivity, high absorption capacity, lengthy availability time, and low cost. Each adsorbent has different properties, such as its active surface, pore size, distribution quality, and surface functional groups^34^. Furthermore, in the context of a circular economy and sustainability, scientists around the world are looking for low-cost adsorbents since agricultural wastes are available as resource materials requiring less natural processing^33^. In addition, compared to commercial adsorbents, the sources are more abundant, such as corn cobs, sugar cane bagasse, walnut shells. sunflower, peanut shells, coffee bean hulls, coffee residue, neem leaves, palm flowers, coconut pith, wheat hulls, mango seeds, plum seeds, jasmine flowers, orange peel, and rice husks have been reported so far^33–35^.

Consequently, the study investigated the adsorption capacity of eggshell (ES), eggshell membrane (ESM), eggshell with the membrane (ESWM), and calcined eggshell (CES) as a green source of adsorbents through the time, metal (copper) concentration, adsorbent dose, temperature, and pH, followed by the RSM to evaluate the optimum adsorption conditions. This study's primary goal was to help the transition to a circular economy and minimize the hazardous impact of waste. Thus, the work and findings of this study can serve as a guide for the manufacturing industry to treat wastewater containing metal ions in an effective and environmentally friendly manner.

## Materials and methods

### Materials

An on-campus cafeteria provided the eggshells used in this study (Taichung Taiwan). Cupric sulfate was purchased from Shimakyu Pharmaceutical Co. (Japan). Ammonium hydroxide was purchased from Hayashi Pure Pharmaceutical Co. (Japan). Sodium hydroxide and hydrochloric acid were purchased from the Union Chemical Plant Co. (Hsinchu, Taiwan).

### Preparation of the four types of eggshell samples

The method described by Lu, et al.^36^ was followed with minor modifications. Briefly, first, an eggshell with a membrane (ESWM) was obtained by cleaning the eggshell with fresh water; on the other hand, the eggshell membrane was divided into eggshell (ES) and eggshell membrane (ESM). The above three samples were dried in an oven at 55 °C for 24 h. Each sample was pulverized for 60 s in a grinder and sieved at 60 mesh. Then, for the calcined eggshell (CES), some of the ESWM powders were calcined in an electric furnace at 700 °C for 2, 3, or 4 h (CES1, CES2, CES3) or at 900 °C for 2, 3, or 4 h (CES4, CES5, CES6).

### Determination of adsorbed copper ions

#### Copper sulfate standard curve

The copper ion assignment was performed following the protocol of Idrees, et al.^37^ with minor modifications. In brief, the copper sulfate solution was prepared at a concentration of 100 mg/L and diluted equally to 6.25, 12.5, 25, 50, and 100 mg/L to produce the standard copper sulfate curve. A 5 mL aliquot of copper sulfate solution with a concentration of 100 mg/L was added to 5 mL of 1 N hydroxide to produce copper hydroxide precipitate. Next, the copper hydroxide was dried in an oven at 100 °C and weighed at 0.3290 g. The molecular weight of copper hydroxide is 97.561 g/mol, while the molecular weight of copper is 63.546 g/mol, so the sample contained 0.2143 g of copper ions. Therefore, the copper ion content in 1 mL of 100 mg/L copper sulfate solution was 0.04286 g/mL.

#### Influence of time on the adsorption rate

The methods were performed with modifications according to Djemmoe, et al.^38^ and Ho, et al.^39^. The eggshell samples were weighed at 10 mg, and 6 mL of copper sulfate solution (pH 5.9) was obtained at a 25 mg/L concentration. Next, the eggshells were placed in 15-mL centrifugation tubes in a 25 °C constant temperature water bath (shaking at 100 rpm) for 1, 2, 3, 24, and 48 h; remarkably, the highest adsorption rate of copper ions was observed at a low shaking/stirring speed^38^. This weak force supports the contact of adsorbent and copper ions without breaking the adsorption force. The above solution was filtered with No. 1 filter paper to remove eggshells, and then 6 mL of filtration solution was added with 3 mL of ammonium hydroxide (10 M) and allowed to rest for 5 min. A spectrophotometer measured the absorbance at 610 nm, and the following equation was used:

1 $$Absorption\; rate \left( \% \right) = \frac{{\left( {OD\;610nm \;before \;adsorption - after } \right)}}{OD\;610nm \;before\; adsorption} \times 100$$

#### Measurement of metal concentration

A 6 mL aliquot of copper sulfate solution (pH 5.9) at concentrations of 25, 50, 100, 150, and 200 mg/L was taken, and the procedure and equation described in Section “Influence of time on the adsorption rate” were used. However, the procedure was carried out in a 25 °C constant temperature water bath (shaking at 100 rpm) for 24 h.

#### Influence of dosage on the adsorption rate

A 6 mL aliquot of copper sulfate solution at a concentration of 25 mg/L (pH 5.9) was taken with the abovementioned eggshell groups weighed at 10, 30, 50, 70, and 90 mg. The procedure in Section “Influence of time on the adsorption rate” was carried out in a 25 °C constant temperature water bath (shaking with 100 rpm) for 24 h.

#### Influence of temperature on the adsorption rate

The procedure and equation described in Section “Influence of time on the adsorption rate” were used, but and procedure was performed in a 25, 35, 45, and 55 °C constant temperature water bath (shaking at 100 rpm) for 24 h.

#### Influence of pH value on the adsorption rate

Taking a 6 mL aliquot of 25 mg/L copper sulfate solution of different pH values (3, 4, 5, 5.9, and 7), the procedure and equation described in Section “Influence of time on the adsorption rate” were used, but the procedure was performed in a 25 °C constant temperature water bath (shaking at 100 rpm) for 24 h.

### Experimental design of RSM

The experiment uses a three-factor, three-variable Box- Behnken test design^40^ to investigate the optimum adsorption conditions for the adsorption of copper ions by eggshell samples such as ESWM, ES, ESM, and CES4. The variables included: adsorption time, metal concentration, and adsorbent dose.

### Statistical analysis

All measurements were performed in triplicate. The values in the tables and figures represent the triplicate measurements’ mean ± standard deviation. The statistical significance of differences among means was evaluated using one-way ANOVA stands for analysis of variance and Duncan’s multiple range tests at a significance level of 0.05.

## Results and discussion

### Effect of time on copper ion adsorption

**Figure 1:**
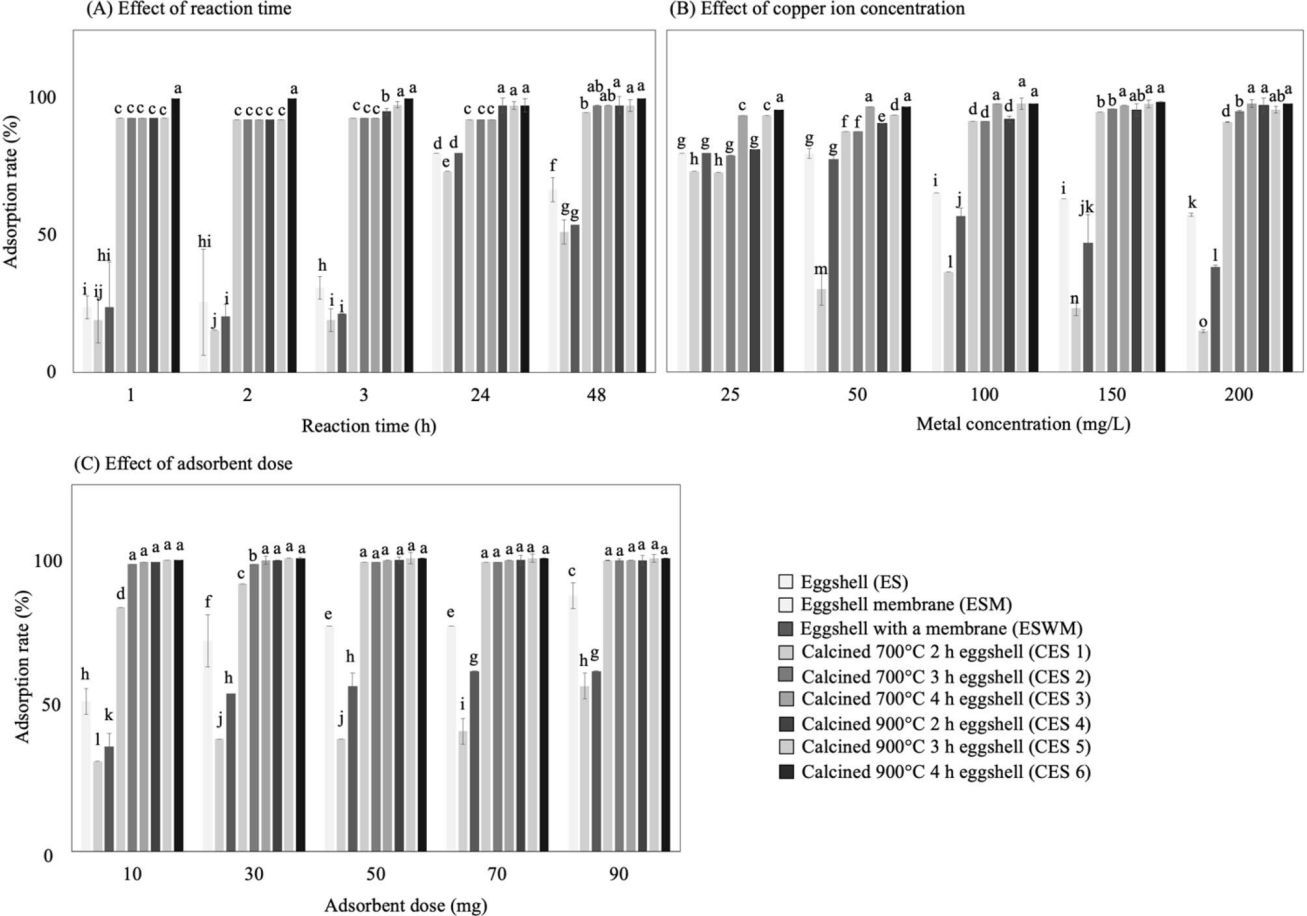
The adsorption rates of differently treated eggshells were influenced by single factors (**A**) reaction time, (**B**) copper ion concentration, and (**C**) dose of adsorbent, respectively.

The effect of time on the adsorption of aqueous copper ions by eggshell samples (ES, ESM, ESWM, and CES) is shown in Fig. 1A. The adsorption rates of ES, ESWM, and ESM were 23.8, 23.8, and 19.1%, respectively, for 1 h; 66.7, 53.9, and 51.3%, respectively, for 48 h; and 80.0, 80.0 and 73.3%, respectively, for 24 h. No significant difference was observed for ES at 24 and 48 h. For ESM and ESWM, a significant difference was observed at 24 h (*p* < 0.05). Hosseini, et al.^15^, Mezenner and Bensmaili^41^ were showed that adsorption involves surface reactions, and with increasing time, the adsorption sites on the adsorbent surface gradually decrease. The effect of surface repulsion prevents adsorption by residual empty adsorption sites on the adsorbent surface. In contrast, adsorption occurs rapidly at the initial stage and slows down as the adsorption sites decrease; thus, the time affected the adsorption rate^15^. A study was conducted on the adsorption of copper ions by rice hulls at a time of 20–180 min, a metal concentration of 100 mg/L, an adsorbent dose of 0.03 g, and a pH range of 2–6; the initial adsorption was rapid and slowed down as the number of adsorption sites decreased^19,42^, which is consistent with the results of this study. However, it was reported that with the positive correlation between the contact time and the adsorption rate of some heavy metals, predominantly in the case of the heavy metal adsorbents, enough sites were available for the metal ions to combine with the eggshells, thus leading to high absorption^7^. The process of eggshells adsorbing Ni^2+^ is reported to rapidly reach equilibrium within 80 min and follow pseudo-second-order kinetics due to the contribution of intraparticle diffusion^6^. In another study, the linear relationships of the pseudo-first-order and pseudo-second-order kinetics of Pb^2+^ and Cd^2+^ ion adsorption were identified as belonging to the intraparticle diffusion model^15^. Hence, the intraparticle diffusion model represents another kinetic model widely used to study the kinetic behavior and explain the mechanisms of pollutant adsorption processes^15,43^.

### Effect of concentration on copper ion adsorption

The initial ion concentration of an aqueous solution has a prominent effect on the adsorption process, as an increase in this concentration leads to an increase in the ion load of the adsorbed species^43^. The adsorption rates of ES, ESWM, and ESM decreased with increasing metal concentrations, and the adsorption rates of ES and ESWM were higher than that of ESM at metal concentrations of 25, 50, 100, 150, and 200 mg/L (Fig. 1B). The adsorption effect of ES was the best, followed by ESWM and then ESM, with significant differences (*p* < 0.05). The adsorption rates of ES, ESWM, and ESM at a metal concentration of 25 mg/L were 80.0, 80.0, and 73.3%, respectively. No significant difference was found between ES and ESWM at a metal concentration of 25 and 50 mg/L. In contrast, ESM at 25 mg/L showed a significant difference (*p* < 0.05), and the adsorption rates at a metal concentration of 200 mg/L were 57.5, 38.4, and 14.9%, respectively. The ESM adsorption rate was lower than the ES and ESWM rates because of the protein composition of ESM, which was blocked by the increased metal concentration and resulted in a poorer adsorption rate. Arami, et al.^44^ showed that the adsorption rate of aqueous Red 23 (DR23) and Direct Red 80 (DR80) by orange peel decreased with the dye concentrations because the total adsorption sites of the orange peel were within the stationary dose of the cell membrane. Thus, the adsorption rate of the adsorbent in an inactive quantity decreases with increasing target matter concentration. Therefore, the initial concentration of eggshell adsorbent decreases the adsorption rate of phosphate, possibly due to the fewness of available adsorption sites under the condition of a specific dosage of eggshell, and the adsorption rate was decreased^19,41^. Kalmykova, et al.^45^ observed that the adsorption rate of aqueous metal on *Sphagnum* peat was 97.3% for a cadmium concentration of 100 ppb and decreased to 86.6% when the cadmium concentration increased to 5000 ppb, which caused the adsorption sites to reach saturation. Another study reported that adsorption efficiency decreased significantly when the initial concentration of ions increased from 10 to 80 mg/L; the possible reasons were the saturation of the active sites on the adsorption surface and the repulsive electrostatic force between the metal ions on the adsorption surface and the aqueous solution^15^. Thus, the metal ions in the aqueous solution could not bind to the adsorption sites, which led to a decrease in the adsorption rate at high metal concentrations.

### Effect of adsorbent dosage on copper ion adsorption

The adsorption of all studied metal ions was reported to be proportional to the dosage of the adsorbent; as implied by many researchers, this finding is due to the increased contact surface area and the consequent increase in the vacant adsorption sites^19^. The study of the effect of adsorbent dose on copper ion adsorption by eggshells showed that the adsorption rates were 51.3, 35.9, and 30.8% for 10 mg of the adsorbent ES, ESWM, and ESM, respectively (Fig. 1C). However, an adsorbent dose of 90 mg showed adsorption rates of 87.2, 61.5, and 56.4%, respectively, and the adsorption rates correlated with the adsorbent dose. Predictably, further increasing the amount of adsorbent did not significantly improve adsorption, as equilibrium was established between the bound and free Cu ions. In contrast, the amount of adsorbent increased globally, and the Cu ion adsorption percentage increased^38^. Vijayaraghavan, et al.^46^ showed that the adsorption rate of copper ions onto crab shells increased with the dosage of adsorbent because of the increase in adsorption sites, so the dosage of adsorbent was a factor affecting the adsorption rate. In addition, it has been suggested that the adsorption efficiency of fluorine would be improved in water at elevated eggshell dosages; in contrast, the defluorination process was dominated by multilayer chemical adsorption^20^.

### Effect of reaction temperature on copper ion adsorption

In the case of high-temperature reactions, the energy of the system caused the metal ions to adhere to the eggshell powder surface more rapidly^43^. The temperature (Fig. 1D) had no significant effect on the adsorption rate of copper ions on ES. In contrast, the adsorption rates of ESWM and ESM increased significantly from 25 to 55 °C. The best adsorption rate of ES at 55 °C was 85.4%, and the adsorption rate did not significantly increase with increasing reaction temperature. Interestingly, the maximum adsorption rates of ESWM and ESM at 55 °C were 81.3 and 56.3%, respectively, correlated with the reaction temperature. The adsorption rate of phosphate on iron hydroxide eggshells has been shown to increase with temperature^41^. Thus, the reaction temperature is a factor affecting the adsorption rate.

### Effect of the pH value on copper ion adsorption

**Figure 2:**
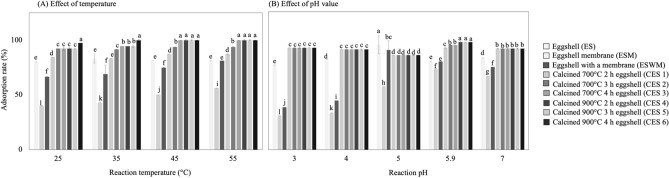
The adsorption rates of differently treated eggshells were influenced by single factors (**A**) reaction temperature and (**B**) pH of the reaction, respectively.

The pH is well known for significantly affecting the absorption of metal ions. Therefore, the ability of H^+^ and other cations to compete for the surface of an adsorption site will be affected by solution pH^15,38,43^. Regarding the effect of pH on the adsorption of copper ions by eggshells (Fig. 1E), the adsorption rates of 76.9, 38.5, and 30.8% were obtained for ES, ESWM, and ESM at pH = 3, respectively, and 83.3, 75, and 66.7% at pH = 7, respectively. The highest adsorption rates were 95.2 and 90.9% for ES and ESWM at pH = 5, respectively. ESM had the highest absorption rate of 73.3% at pH = 5.9. Notably, the surface of the adsorbent became negatively charged with increasing pH, thus facilitating the adsorption of copper, while free copper ions could bind to the adsorption sites by mechanisms other than physical adsorption^38^. Eggshells at pH = 6.0 are reported to remove heavy metal ions (Co^3+^, Zn^2+^, Hg^2+^, Pb^2+^) from tannery wastewater by physical adsorption with an efficiency of approximately 99%^43^. The carbonate ions produced by ES^47^ did not interact with them, which caused a significant decrease in the adsorption rate of ES, as well as ESWM and ESM, at low pH. Several studies have revealed that pH is an influencing factor for the adsorption process of metals in aqueous solutions, as it affects the degree of ionization and surface properties of the adsorbent^15,48^. It has been suggested that the ability of eggshells to adsorb metals is enhanced by increasing the solution pH^47^. Moreover, the eggshells were dissolved at weakly acidic pH^7,20,47^, which was one of the reasons for the decrease in the adsorption rate^48^. The eggshell hydrolysis reaction occurs in aqueous solutions, the system becomes more alkaline, and the amount of OH ions increases, providing a substantial advantage by naturally modifying the solution pH without any other chemicals^49^. Furthermore, a significant decrease in the absorption rate of the ESM was observed at pH = 5.9, which was attributed to the formation of copper hydroxide precipitate by modifying the pH with 0.1 N sodium hydroxide, which was related to the shape of a more insoluble ion. Above pH = 5.9, the copper metal ions probably produced copper hydroxide precipitate, which caused a decrease in the adsorption rate. However, no significant difference in the absorption rate of the eggshells with membrane was observed under the above conditions (Fig. 2).

### Effect of eggshell calcination temperature on adsorption

**Figure 3:**
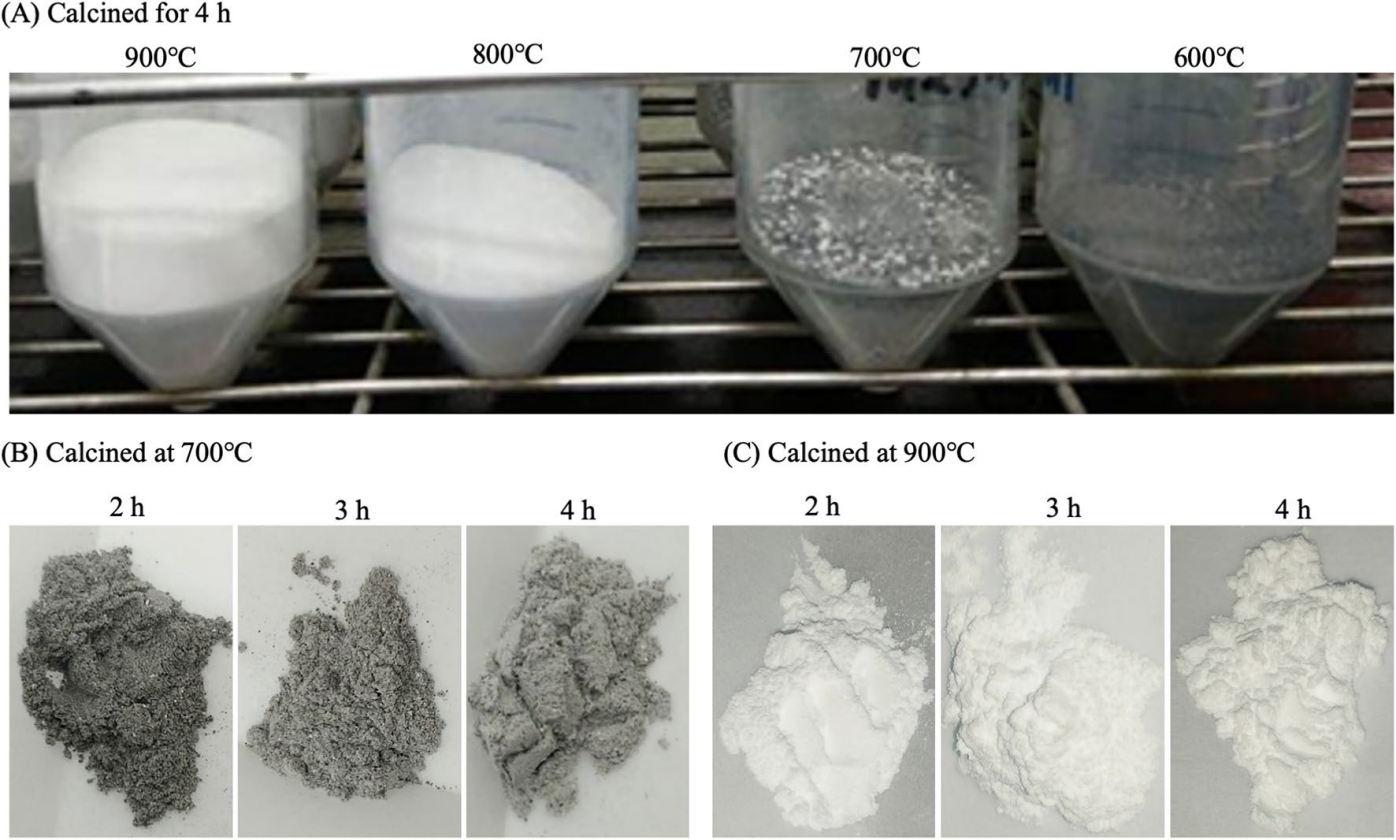
The appearance of eggshells that were calcined under different conditions. (**A**) Calcined for 4 h at different temperatures; (**B**) calcined for 2–4 h at 700 °C; and (**C**) calcined for 2–4 h at 900 °C.

The powder was black and composed of calcium carbonate when calcination was performed at 600 °C for 4 h, and it was off-white when calcined at 700 °C for 4 h, with a surface distribution of calcium carbonate calcined to calcium oxide, whereas beneath the surface the composition remained as calcium carbonate (Fig. 3A). Köse and Kıvanç^50^ showed that the powder of eggshells incinerated at 800 °C for 4 h was white, and the original calcium carbonate was incinerated to calcium oxide. The powder calcined at 900 °C for 4 h was white and composed of calcium oxide. The appearance of the eggshells calcined at 700 °C for 2, 3, and 4 h is shown in Fig. 3B. The grayish powder showed that the eggshells were not completely calcined, while the white powder was the completely calcined calcium oxide. Another CES treated at 900 °C for 2, 3, and 4 h has a white appearance in Fig. 3C, where the white powder is the calcium oxide obtained from the complete calcination.

The study of the effect of time on the adsorption of copper ions from calcined eggshells (Fig. 1A) showed that the adsorption rates of CES1, CES2, and CES3 were 92.9% at 1 h of adsorption time. The maximum adsorption time was 48 h, which resulted in 94.9, 97.4, and 97.4% adsorption rates, respectively. The lowest adsorption time of 2 h showed an adsorption rate of 92.3% for all three groups of CESs. The adsorption rates of CES4, CES5, and CES6 were 92.9, 92.9, and 100.0% at 1 h, respectively. The maximum adsorption at 48 h was 97.4, 97.4, and 100.0%, respectively. Notably, no statistically significant differences in adsorption time were observed from 1 to 48 h for groups CES1-6, which showed the stable adsorption of all copper ion aqueous solutions. However, the adsorption rate was rapid at the initial stage of adsorption, followed by a decrease in adsorption sites as time increased, in agreement with the results in Hassan, et al.^43^.

The study of the effect of metal concentration (25, 50, 100, 150, and 200 mg/L) on the adsorption of copper ions by calcined eggshells (Fig. 1B) showed that a metal concentration of 25 mg/L resulted in the best adsorption rate of CES3 of 93.8%, followed by CES2 at 79.2% and CES1 at 72.9%. In contrast, the best adsorption rate of CES6 was 95.8% at a metal concentration of 25 mg/L, followed by CES5 at 93.8% and CES4 at 81.3%. For a metal concentration of 150 mg/L, the adsorption rates of CES4, CES5, and CES6 were 97.5, 95.9, and 98.1%, respectively, and no significant difference was observed at a metal concentration of 200 mg/L. This result may be due to the available adsorption sites being limited under a specific dosage of eggshell adsorbent, which will not increase with the metal concentration, thereby leading to a decrease in the adsorption rate. Kalmykova, et al.^45^ found that at high concentrations of metal ions, the adsorbent reaches saturation at the adsorption sites. The metal ions in the aqueous solution could not bind with vacant adsorption sites, which decreased its adsorption rate at high metal concentrations. Therefore, it was hypothesized that the adsorption sites for a specified dose of CESs were limited, decreasing as the metal concentration increased. However, CES1-6 showed effective adsorption at a metal concentration of 200 mg/L, which means that calcined eggshells still had many vacant adsorption sites to adsorb copper ions, which caused the adsorption rate of calcined eggshells to increase with the metal concentration. No significant change was reported in the eggshell adsorption rate with increasing HNO_3_ concentration until it exceeded 1M^48^. As we all know, eggshell is mainly composed of calcium carbonate (CaCO_3_) before calcination^51^, with a typical irregular crystal structure with tiny pores, and this structure implies that the adsorption of metal ions on the surface of uncalcined eggshells may be due to the physical adsorption mode^43^. However, calcined eggshells are primarily composed of CaO, whose network has smaller pores due to CO_2_ removal and larger porosities due to aggregation into interconnected skeletal structures. Therefore, the calcined eggshell had a better metal adsorption capacity. On the other hand, it has been estimated that CES6 was calcined at 900 °C for 4 h. The calcination of the surface of CES6 was relatively complete, which resulted in the best adsorption rate for copper metal.

In contrast, CES1, calcined at 700 °C for 2 h, showed some white calcium oxide on the surface of the powder, while the bottom layer was not wholly calcined, with the calcium carbonate causing the low adsorption rate. Significantly, temperatures influence the completeness of the calcination, and the calcination temperature might have a more significant effect than the time since the adsorption rate was significantly higher at a calcination temperature of 900 °C for 2 h (CES 4) than at 700 °C (CES2). In comparison with the above results (Fig. 1B), overall, the adsorption rate of the calcined eggshells was higher than the uncalcined ones.

The adsorption of copper ions by calcined eggshells (Fig. 1C) showed rates of 83.2, 98.0, and 98.7% for CES1, 2, and 3, respectively, with a 10 mg adsorbent dose. In comparison, an adsorption rate of 99.3% for all three groups was achieved by increasing the amount to 90 mg. A significant increase (*p* < 0.05) was observed for the CES1 dose from 10 to 50 mg, which could be attributed to an increase in the number of adsorption sites on the surface as the dosage increased, which suggests that the adsorption rate of CES1 showed a current correlation with the dosage. However, no significant difference was observed between CES2 and 3 in the effects of the adsorbent. This result means that the 10 mg adsorbent of CES2 and 3 completely adsorbed the copper ions. Similarly, the adsorption rate of CES1 was significantly different (*p* < 0.05) compared to the above two groups at 10 and 30 mg doses. A possible reason for the difference might be that the lack of calcination time led to incomplete calcium oxide conversion.

On the other hand, the adsorption rates of CES4, 5, and 6 were 98.7, 99.3, and 99.3%, respectively, at a 10 mg of adsorbent dose and 100.0% at a 90 mg adsorbent dose. Meanwhile, no significant difference was observed in the adsorption rate as the amount increased to 90 mg, which implies that only 10 mg was necessary for CES4, 5, and 6 to adsorb the copper ions specified in this study completely. In addition, compared to the adsorption rate results for the same factor above (Fig. 1C), calcined eggshells showed a better performance.

The study of the adsorption of copper ions by calcined eggshells at various temperatures (Fig. 2A) showed that at 25 °C, CES1 and 6 differed (*p* < 0.05), being 84.6 and 97.4%, respectively, while CES2, 3, 4, and 5 were 92.3%. However, the adsorption rates of CES1 and 2 were 87.5 and 93.8% at 55 °C, respectively, and the remaining four groups of CES3-6 were 100%. CES1, 2, and 3 showed a significant increasing trend (*p* < 0.05) at temperatures from 35 °C to 45 °C. The endothermicity of adsorption depends on the temperature, and the results of Flores-Cano, et al.^47^ agreed with this study. CES4 and 5 showed a significant increase (*p* < 0.05) from 25 to 55 °C, and the possible reasons are the same as above. The adsorption rate of CES6 was 100.0% at reaction temperatures of 35 to 55 °C, with no significant difference. The adsorption of anionic dyes from solution was studied using crushed eggshells, in agreement with the phenomenon found in this study^52^. Another influencing factor in the adsorption process is the temperature, which affects the viscosity of the solution; on the other hand, adsorption depends on the different thermal responses of the materials. A study using the nanocomposite of eggshell/starch/Fe_3_O_4_ to adsorb Cd^2+^ and Pb^2+^ ions found that adsorption was exothermic and spontaneous, as the degree of the spontaneity of the reaction decreased with increasing temperature^15^.

Figure 2B shows the results of the adsorption of copper ions by calcined eggshells at different pH levels. CES1-6 showed a sorption rate of 92.3% at pH 3 and 91.7% at pH 7. The pH 5.9 adsorption rates were 92.3, 94.9, and 94.9% in the case of CES1, 2, and 3, respectively, and 97.4% for CES4-6. These results indicate that calcined eggshells could adsorb copper ions at pH 3 to 7. Compared with the above results (Fig. 2B), the calcined eggshells had good neutralization in acidic wastewater. The calcined eggshell can remove heavy metals with a neutralization ability in strong acidic wastewater. Thus, the adsorption rate under acidic aqueous solution was higher for calcined eggshells than for uncalcined ones.

### Optimal conditions obtained from RSM

In this study, to investigate the interaction effects of 4 eggshells such as ESWM, ES, ESM, and CES4 (900 °C, 2 h) with each other, RSM was used. The Box- Behnken design (BBD) obtained the optimal adsorption conditions with three factors and three levels of experimental design^40^. The RSM covers a series of multifactor experiments designed and statistically validated to obtain optimum operating conditions. Thus, using a minimum number of trials to obtain an optimal response while calculating the relationship between the various citation factors is possible^53^. The experimental conditions of the time, metal concentration, and adsorption dose were used as independent variables. The reaction temperature and pH were set at 25 °C and 5.9, respectively, as the reaction (adsorption) conditions for considering the cost of future applications in wastewater treatment. According to the literature reported^54^, ANOVA was performed to check the statistical significance of the regression terms.

#### Optimal conditions for ES adsorption

The experimental conditions of the reaction time (12–36 h), metal concentration (25–75 mg/L), and adsorption dose (1.0–2.0 g) were used as independent variables (Table 1). A reaction temperature of 25 °C and pH of 5.9 was set as the reaction (adsorption) conditions for considering the cost of future applications in wastewater treatment. BBD experiments designed the above independent variables to obtain the combinations of 15 groups of adsorption conditions (Table 2). The results showed that the lowest and highest adsorption rates were 30.8% and 93.3% for groups 7 and 1, respectively. Otherwise, the adsorption rates ranged from 42.9 to 84.8%. The above results were analyzed using Statgraphics 19 (Statgraphics Technologies, Inc., The Plains, Virginia, USA) by obtaining the individual parameters (Table 3 and A1) with the quadratic regression equation shown below:

2 $$\begin{aligned} {\text{Y }} = & - {53}.{625} + 0.{71}00{\text{69A}} - 0.{2}0{\text{1667B}} + {154}.{\text{525C }} \\ & - 0.0{616}0{\text{3A}}^{{2}} + 0.000{\text{5AB}} + {1}.{\text{3125AC}} + 0.00{\text{728667B}}^{{2}} \\ & + \, 0.0{\text{72BC}} - {65}.0{\text{833C}}^{{2}} \\ \end{aligned}$$

A: Reaction time.B: Metal concentration.C: Adsorption dose.

**Table 1:** Parameter range of the 3-level-3-factor Box–Behnken design (BBD) of adsorption rate from four different eggshells.

Factor	Eggshell (ES)			Eggshell membrane (ESM)			Eggshell with a membrane (ESWM)			Calcined eggshell (CES4)		
	Coded level of factor											
	− 1	0	1	− 1	0	1	− 1	0	1	− 1	0	1
Reaction time (h)	12	24	36	12	24	36	12	24	36	20	40	60
Concentration (mg/L)	25	50	75	25	50	75	25	50	75	100	150	200
Adsorption dose (g)	1	1.5	2	100	150	200	1	1.5	2	1	10	19

A reaction temperature of 25 °C and pH 5.9 were used as fixing factors.

**Table 2:** Box–Behnken design (BBD) for adsorption rate of four different eggshells in 3-level-3-factor.

Treatment	Eggshell (ES)				Eggshell membrane (ESM)				Eggshell with a membrane (ESWM)				Calcined eggshell (CES4)			
	Coded level of factor			Y (%)	Coded level of factor			Y (%)	Coded level of factor			Y (%)	Coded level of factor			Y (%)
	Reaction time (h)	Concentration (mg/L)	Adsorption dose (g)	Adsorption rate	Reaction time (h)	Concentration (mg/L)	Adsorption dose (g)	Adsorption rate	Reaction time (h)	Concentration (mg/L)	Adsorption dose (g)	Adsorption rate	Reaction time (h)	Concentration (mg/L)	Adsorption dose (g)	Adsorption rate
1	0 (24)	1 (75)	− 1 (1.0)	93.3	1 (36)	0 (50)	− 1 (100)	40.9	0 (24)	0 (50)	0 (1.5)	26.3	0 (40)	− 1 (100)	− 1 (1.0)	98
2	− 1 (12)	− 1 (25)	0 (1.5)	61.5	1 (36)	− 1 (25)	0 (150)	35.7	− 1 (12)	− 1 (25)	0 (1.5)	22.2	− 1 (20)	0 (150)	− 1 (1.0)	97.3
3	− 1 (12)	1 (75)	0 (1.5)	73.7	0 (24)	0 (50)	0 (150)	47.4	− 1 (12)	0 (50)	− 1 (1.0)	57.9	1 (60)	0 (150)	1 (19.0)	100
4	1 (36)	− 1 (25)	0 (1.5)	66.7	0 (24)	0 (50)	0 (150)	42.1	0 (24)	− 1 (25)	− 1 (1.0)	11.1	0 (40)	0 (150)	0 (10.0)	98.6
5	− 1 (12)	0 (50)	1 (2.0)	50	0 (24)	0 (50)	1 (200)	47.4	0 (24)	0 (50)	0 (1.5)	31.6	0 (40)	− 1 (100)	1 (19.0)	100
6	− 1 (12)	0 (50)	− 1 (1.0)	66.7	0 (24)	1 (75)	1 (200)	62.5	0 (24)	0 (50)	0 (1.5)	36.8	1 (60)	0 (150)	− 1 (1.0)	98.6
7	0 (24)	− 1 (25)	1 (2.0)	30.8	1 (36)	0 (50)	1 (200)	50	0 (24)	1 (75)	1 (2.0)	64.9	1 (60)	1 (200)	0 (10.0)	98.6
8	0 (24)	0 (50)	0 (1.5)	80	0 (24)	− 1 (25)	− 1 (100)	55.6	1 (36)	1 (75)	0 (1.5)	54.1	− 1 (20)	0 (150)	1 (19.0)	100
9	1 (36)	1 (75)	0 (1.5)	79.5	0 (24)	− 1 (25)	0 (150)	22.2	1 (36)	0 (50)	1 (2.0)	63.2	0 (40)	1 (200)	1 (19.0)	100
10	0 (24)	0 (50)	0 (1.5)	64	0 (24)	1 (75)	− 1 (100)	43.8	− 1 (12)	1 (75)	0 (1.5)	62.2	1 (60)	− 1 (100)	0 (10.0)	100
11	1 (36)	0 (50)	− 1 (1.0)	33.3	1 (36)	1 (75)	0 (150)	57.9	− 1 (12)	0 (50)	1 (2.0)	68.4	0 (40)	0 (150)	0 (10.0)	97.3
12	1 (36)	0 (50)	1 (2.0)	48.1	− 1 (12)	− 1 (25)	0 (150)	36.4	0 (24)	1 (75)	− 1 (1.0)	62.2	0 (40)	1 (200)	− 1 (1.0)	86.7
13	0 (24)	− 1 (25)	− 1 (1.0)	42.9	− 1 (12)	0 (50)	− 1 (100)	34.8	0 (24)	− 1 (25)	1 (2.0)	11.1	− 1 (20)	− 1 (100)	0 (10.0)	100
14	0 (24)	0 (50)	0 (1.5)	80	− 1 (12)	0 (50)	1 (200)	47.8	1 (36)	− 1 (25)	0 (1.5)	11.1	− 1 (20)	1 (200)	0 (10.0)	99
15	0 (24)	1 (75)	1 (2.0)	84.8	− 1 (12)	1 (75)	0 (150)	70.3	1 (36)	0 (50)	− 1 (1.0)	42.8	0 (40)	0 (150)	0 (10.0)	100

**Table 3:** Optimum conditions and the predicted adsorption yield value of the response at the optimum conditions.

Factor	Eggshell (ES)				Eggshell membrane (ESM)				Eggshell with a membrane (ESWM)				Calcined eggshell (CES4)			
	Low	High	Optimum conditions	Modified condition	Low	High	Optimum conditions	Modified condition	Low	High	Optimum conditions	Modified condition	Low	High	Optimum conditions	Modified condition
Reaction time (h)	12	36	21.4621	21.5	12	36	12	12	12	36	12	12	20	60	20.0703	20
Concentration (mg/L)	25	75	75	75	25	75	74.582	75	25	75	74.9916	75	100	200	179.764	180
Adsorption dose (g)	1	2	1.44505	1.445	100	200	200	200	1	2	2	2	1	19	19	19
Adsorption (%)	95.8064				75.3847				80.6				100			

**Figure 4:**
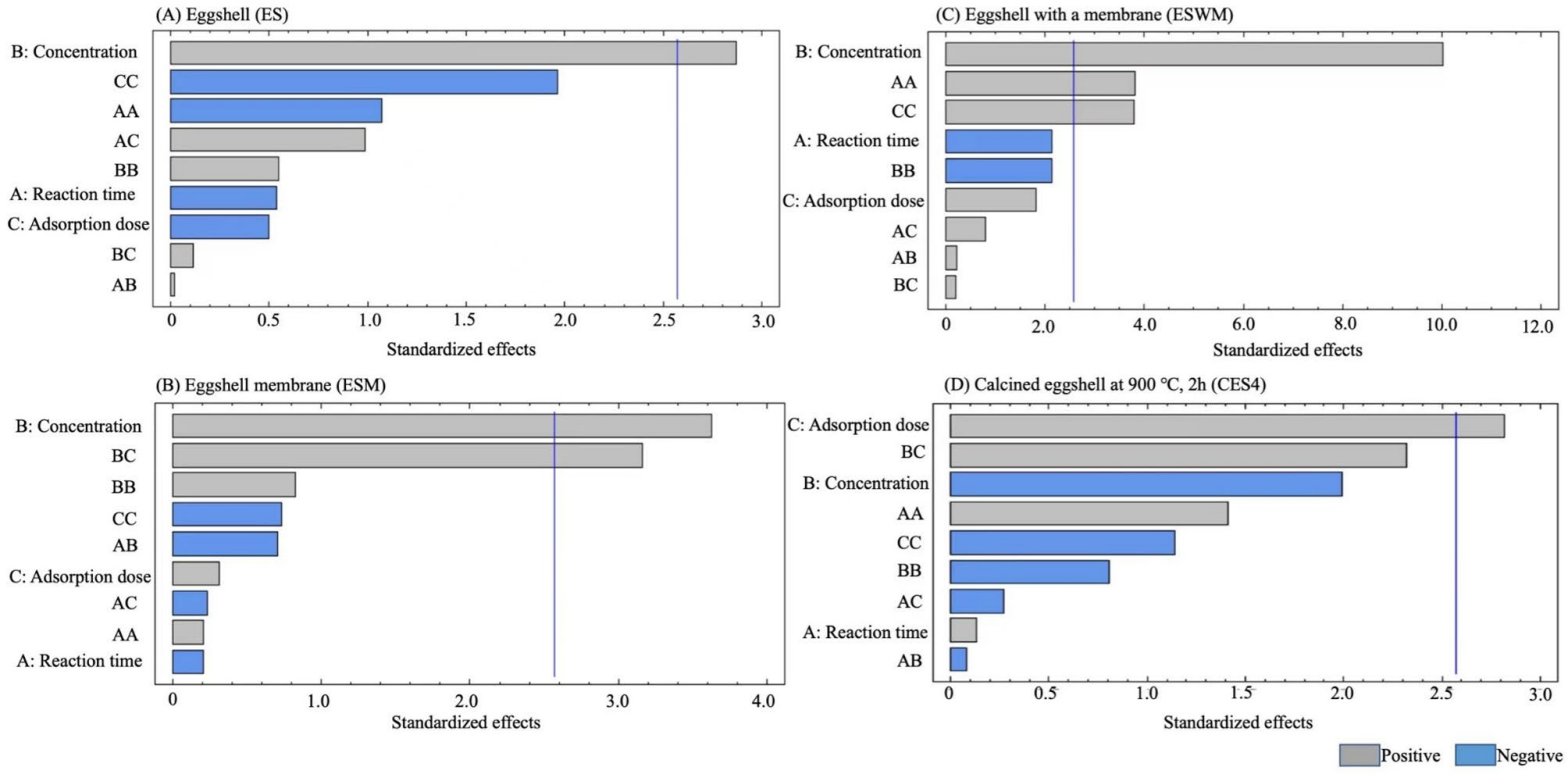
Pareto chart standardized effects for the adsorption of 4 different eggshells in copper ion solution. (**A**) Eggshell (ES); (**B**) eggshell membrane (ESM); (**C**) eggshell with a membrane (ESWM); (**D**) calcined eggshell at 900 °C, 2 h (CES4).

The ANOVA analysis (Table A2) showed that among the independent factors, only one effect was significant (*p* < 0.05), whereas only the primary item of concentration significantly affected the copper ion absorption rate. The other factors and interactions were not significantly different (*p* > 0.05). R^2^ was 75.0689%, which means that the quadratic regression equation of this study correlated well with the experimental results of the reaction surface. Moreover, the mathematical model constructed from the results was meaningful and valid, accurately describing the results presented by the response surface. In addition, the Pareto chart (Fig. 4A) analyzed the primary and secondary effects, which showed that the primary term of metal concentration (B) exceeded the significant line, indicating that the metal concentration had a significant impact on the adsorption rate of copper ions. The primary term of reaction time, the primary period of adsorbent dose, and the secondary terms of reaction time and adsorbent dose have adverse and significant effects on the adsorption rate of copper ions, which implies that the design of the center point of the downward modification time and dose will benefit the increase in adsorption rate.

The adsorption time and metal concentration results on copper metal ion adsorption at a dose of 1.5 g of adsorbent (CES4) (Figure A1, A) showed that the adsorption rate increased with metal concentration, with a maximum adsorption rate occurring at 65 to 75 mg/L. In addition, the maximum adsorption rate occurred at a 50 mg/L metal concentration with an adsorption time of 20 to 24 h and an adsorbent dose of 1.4 to 1.6 g (Figure A1, A). The maximum adsorption rate was observed between 65 and 75 mg/L when the adsorption time was 24 h, which correlated with the metal concentration (Figure A1, A), and the highest adsorption rate occurred between 1.4–1.6 g of adsorbent dose. A longer reaction time did not lead to a higher adsorption rate. As previously reported, the adsorption rate tends to balance with the decrease in the adsorption sites of the adsorbent, leading to difficulty of adsorption. According to the above results, the adsorption sites for metal ions on eggshells were limited. Nonetheless, the adsorption rate tended to increase with the metal concentration, which means that adsorption might continue at unsaturated adsorption sites at a specific dose of adsorbent. In this study, an adsorbent dose of 1.445 g was calculated using the equation, which resulted in a 92.7% adsorption rate by triplicate experiments. However, the relationship between the adsorbent dose and the adsorption rate was not linear. The optimal adsorption conditions for the reaction surface and the predicted best adsorption rates are shown in Table 4. The optimal adsorption conditions were a 21.5 h reaction time, 75 mg/L metal concentration, and 1.445 g adsorbent dose. The predicted adsorption rates of copper ions under the above conditions ranged from 74.0 to 95.8%, and the subsequent triplicate experiments confirmed that an adsorption rate of 92.7% (Table 4) was statistically insignificant (*p* > 0.05), which means that the above mathematical model is accurate and valid.

**Table 4:** Verification tests for the fitted model of optimum adsorption.

Group	Variable: Adsorption rate												
	N	Lower	Mean	Upper	Lower	Std	Upper	Std	Min	Max	DF	*t* value	Pr $>\left|t\right|$
		CL		CL	CL Std	Dev	CL Std	Err					
		Mean		Mean	Dev		Dev						
Eggshell (ES)	3	89.7	92.7	95.7	0.62	1.2	7.54	0.69	91.5	93.9	2	4.47	0.05
Eggshell membrane (ESM)		58.4	67	75.6	1.8	3.46	21.74	2	63.7	70.6	2	4.21	0.05
Eggshell with a membrane (ESWM)		58.4	67	75.6	1.8	3.46	21.74	2	63.7	70.6	2	4.21	0.05
Calcined eggshell (CES4)		64.7	76.4	88.1	2.45	4.71	29.62	2.72	71.9	81.3	2	1.54	0.26

#### Optimal conditions for ESM adsorption

In this study, in which ESMs were used as an adsorbent, the reaction time (12–36 h), metal concentration (25–75 mg/L), and adsorbent dose (100–200 mg) were considered independent variables (Table 1), and 15 combinations of these variables were obtained from BBD (Table 2). As Table 2 shows, the adsorption rate of group 9 was 22.2%, the lowest among the 15 groups, with an average absorption rate of 42.1–62.5%. A maximum adsorption rate of 93.3% was obtained for group 15 adsorption conditions. The parameters obtained from the Statgraphic software analysis (Table 3 and A1) and the quadratic regression equation were as follows:

3 $$\begin{aligned} {\text{Y}} = & {72}.{8875} + 0.{\text{382639A}} - {1}.{\text{47333B}} - 0.0{\text{85C}} \\ & + 0.00{6221}0{\text{6A}}^{{2}} - 0.00{\text{975AB}} - 0.00{\text{1625AC}} \\ & + 0.00{\text{567333B}}^{{2}} + 0.0{1}0{\text{42BC}} - 0.00{\text{126167C}}^{{2}} \\ \end{aligned}$$

A: Reaction time.B: Metal concentration.C: Adsorption dose.

The effect of reaction time, metal concentration, and adsorbent dose on the adsorption rate of adsorbed copper ions was investigated using ANOVA analysis (Table A3), and only one term (B), the effect of metal concentration, was significant among the factors (*p* < 0.05). The interaction between metal concentration and adsorbent dose significantly affected the adsorption rate. The other primary, secondary, and interaction terms were insignificant (*p* > 0.05). R^2^ was 83.4266%. The Pareto chart analysis of the primary and secondary effects (Fig. 4B) showed that the primary terms of concentration, metal concentration, and adsorbent dose interaction exceeded the significant line. This result means that the above factors significantly affect the adsorption rate of copper ions. Meanwhile, the primary term of time and the secondary term of adsorbent dose had significant negative effects on the adsorption rate of copper ions, which means that a lower center point of time and dose would benefit the adsorption of copper ions. The maximum adsorption rate for ESM was observed at a 150 mg adsorbent dose from 65 to 75 mg/L, and the adsorption rate correlated with the metal concentration (Figure A1, B). At a 50 mg/L metal concentration, the adsorption time was 12 h, and the adsorption rate of the ESM adsorbent dose of 200 mg showed the maximum adsorption rate (Figure A1, B). The adsorption rate was maximized at a metal concentration of 65 to 75 mg/L for 24 h of reaction time, and the adsorption rate tended to increase with the metal concentration and the dose of adsorbent; the maximum adsorption rate was observed at a dose of 200 mg (Figure A1, B). This result means that the adsorption rate was correlated with the adsorption dose. The best adsorption rate of 75.4% was verified using the above equation for an adsorbent dose of 200 mg. The optimum adsorption conditions for RSM were a 12 h reaction time, 75 mg/L metal concentration, and 200 mg adsorbent dose, which resulted in estimated adsorption rates of 60.0- 75.4% (Table 4). As verified by the triplicate experiments, the actual adsorption rate of 67.0% was statistically not significantly different (*p* > 0.05), indicating that no significant difference was observed with the predicted values, and the derived equation was accurate and valid (Table 4).

#### Optimal conditions for ESWM adsorption

The ESWM was used as the adsorbent, and the independent variable conditions were described in Section “Optimal conditions for ESM adsorption” above. The results are shown in Table 1, while BBD obtained for the 15 combinations of adsorption conditions is shown in Table 2. The adsorption rates were 11.1% for groups 4, 13, and 14 and ranged from 11.1 to 64.9% for other groups, with the maximum adsorption rate of 68.4% obtained for the adsorption condition of group 11. Using Statgraphic software analysis, each parameter (Tables 3 and A1) and the quadratic regression equation were obtained as follows:

4 $$\begin{aligned} {\text{Y}} = & 146.225 - 5.60{\text{764A}} + 1.{\text{97783B}} - 162.{\text{275C}} \\ & + 0.0{\text{916956A}}^{2} + 0.00{\text{25AB}} + 0.{\text{441667AC}} \\ & - 0.0{\text{117933B}}^{2} + 0.0{\text{54BC}} + 52.{\text{5167C}}^{2} \\ \end{aligned}$$

A: Reaction time.B: Metal concentration.C: Adsorption dose.

The ANOVA analysis (Table A4) showed a significant effect (*p* < 0.05) with one factor, indicating that the primary term of metal concentration and the secondary terms of reaction time and adsorbent dose significantly affected the adsorption rate of copper ions. Other variables were insignificant (*p* > 0.05) for the primary, secondary, and interaction terms; R^2^ was 96.629%. According to the Pareto chart for the primary and secondary effects (Fig. 4C), the primary term of metal concentration, and the secondary terms of reaction time and adsorbent dose exceeded the significant line, which indicated that these factors had significant effects on the adsorption rate. However, the primary term of reaction time and the second term of metal concentration had significant negative effects on the adsorption rate, indicating that a lower center point of time and metal concentration would favor copper ion adsorption. However, the predicted metal concentration of 75 mg/L for the optimum adsorption rate was higher than the experimental design center point of 50 mg/L. The effect may be due to the significant primary effect of metal concentration. The adsorption rate of ESWM at a 1.5 g adsorbent dose increased with the metal concentration, and the highest adsorption rate was achieved between 65 and 75 mg/L (Figure A1, C). At a 50 mg/L metal concentration, an adsorption time of 12 h and an adsorbent dose of 2.0 mg showed the highest adsorption rate (Figure A1, C). The reaction time was 24 h, and the maximum adsorption rate was achieved at a metal concentration of 65–75 mg/L; moreover, 2.0 g of adsorbent dose had the maximum adsorption rate (Figure A1, C). Triplicate trials obtained an optimum adsorption rate of 76.4% with a predicted dosage of 2.0 g of adsorbent. At the same time, plots of the adsorbed amount of copper ions relative to the variables responding to the surface were shown to understand the interactions between the variables and verify the optimal levels of each variable to achieve the maximum adsorption value^55^. The optimum adsorption conditions for RSM were a 12 h reaction time, 75 mg/L metal concentration, and 2.0 g adsorbent dose (Table 3), which resulted in predicted adsorption rates of 60.0–80.6%. Triplicate trials subsequently verified the actual adsorption rate of 76.4% with no significant difference from the predicted values (*p* > 0.05) (Table 4), which confirmed the validity of the mathematical model in this study Consistent with many studies, the results of the experiment showed remarkable agreement with the measured RSM values^55–57^.

#### Optimal conditions for CES4 adsorption

CES4 (900 °C, calcined for 2 h) was used as the adsorbent, and the three factors of reaction time (20–60 min), metal concentration (100–200 mg/L), and adsorbent dose (1.0–19.0 mg) were used as independent variables (Table 1). The BBD was used to obtain 15 combinations of these variables (Table 2). Table 2 shows that the lowest adsorption rate was 86.7% for group 13. In comparison, the adsorption rates of other groups ranged from 98.0 to 100.0%, with groups 5, 8, 9, 10, 13, and 15 having the highest absorption rate of 100.0%. Using Statgraphic software analysis, the following parameters (Tables 3 and A1) and quadratic regression equation were obtained.

5 $$\begin{aligned} {\text{Y}} = & 105.149 - 0.{\text{317986A}} + 0.0{\text{289722B}} - 0.2440{\text{33C}} \\ & + 0.00{\text{445833A}}^{2} - 0.000{\text{1AB}} - 0.00180{\text{556AC}} \\ & - 0.00040{\text{6667B}}^{2} + 0.00{\text{627778BC}} - 0.0{\text{177984C}}^{2} \\ \end{aligned}$$

A: Reaction time.B: Metal concentration.C: Adsorption dose.

The effect of reaction time, metal concentration, and adsorbent dose on the adsorption rate was investigated using the three variables ANOVA (Table A4), which showed a significant effect (*p* < 0.05) within the factors, indicating that the primary term of the adsorbent dose had a significant effect on the adsorption rate. The other primary, secondary, and interaction terms were insignificant (*p* > 0.05), with an R^2^ of 81.2286%. The Pareto chart analyzes the primary and secondary effects (Fig. 4D). The primary term of the adsorbent dose exceeded the significant line, indicating a significant impact on the adsorption rate of copper ions. The primary and secondary metal concentrations and the secondary adsorbent dose reduce the adsorption rate of copper ions, which means that a lower center point of metal concentration and adsorbent dose would benefit the adsorption rate. The optimum adsorption rate conditions were 180 mg/L for the metal concentration and 19.0 mg for the adsorbent dose, which were higher than the experimental design center points of 150 mg/L and 10.0 mg, respectively due to the interaction effects of reaction time, metal concentration, Fig. 2 and adsorbent dose. For CES4 at a 10.0 mg adsorbent dose, the reaction time was 20–30 min with maximum adsorption rates of 160–180 mg/L metal concentration (Figure A1, D). The maximum adsorption rate was achieved for CES4 at a 150 mg/L metal concentration with a reaction time of 20–30 min and an adsorbent dose of 16.0–20.0 mg (Figure A1, D). The maximum adsorption rate was between 160–180 mg/L for the metal concentration and 16.0–20.0 mg for the adsorbent dose for 40 min of adsorption time in CES4 (Figure A1, D). The maximum adsorption rate was observed at 19.0 mg for the adsorbent dose. The optimum adsorption conditions for RSM were a 20 min reaction time, 180 mg/L metal concentration, and 19.0 mg adsorbent dose (Table 4). However, it was reported that the first stage of metal ion adsorption was rapid, generally completed within 10 min^19^. The estimated adsorption rate of 99.6–100.0% was not significantly different (*p* > 0.05) from the triplicate trial validation of 99.3% (Table 4). It means that the equation derived in this study is accurate and valid while in agreement with the results of other studies^53,57^. According to a study on copper ion absorption by rice hulls, approximately 33% of copper ions were removed within 30 min at pH 4, with a load of 5.0 g^42^. CESs was reported to be effective for heavy metal removal in microalgae hybrid systems; hence, CES is a nontoxic, noncorrosive, and environmentally safe material to handle; as such, it has the potential for broader application within wastewater treatment technologies^13,58^.

## Conclusions

Earth provides many valuable materials, and while some seem unused, their potential for hidden applications has always been found. This study used different parts of discarded eggshells and calcined eggshells for copper ion adsorption measurements. In a single factor, at a reaction time of 24 h, a metal concentration of 25 mg/L, an adsorbent dose of 10 mg, and a temperature of 25 °C, the adsorption rates of ES and ESWM at pH 5 were 95.2 and 90.5%, respectively, while that of ESM at pH 5.9 was 73.3%. However, a good adsorption rate was observed for all groups with CES1- 6. RSM investigated the optimum adsorption rate conditions and showed that the CES2 adsorption rate reached 99.3% at a reaction time of 20 min, which was higher than that of ES (92.7%), ESM (67%), and ESWM (76.4%) at a reaction time of 12–21.5 h. Therefore, the conversion of eggshell waste in large quantities and at low cost into biological adsorbents may be applied to the adsorption of metals in industrial wastewater, which has marginal economic benefits that cannot be ignored. At the same time, it has the dual benefits of being eco-friendly and recycling waste materials. In a practical application of wastewater treatment, the eggshell powder adsorbs higher concentrations of copper ion wastewater; as a first pretreatment, the concentration was reduced to 180 mg/L, followed by a 2nd stage treatment with 99.3% adsorption by calcined eggshells. Eggshells can be easily obtained and are inexpensive; although the abovementioned potential is encouraging, the process increased their manufacturing costs severalfold, which represents a valuable direction for future research, thus contributing to the development of sustainable food systems.

## Supplementary Information

Supplementary Information.

## Data Availability

The datasets used during the current study available from the corresponding author on reasonable request.
