# Supplementary material for: On the removal efficiency of copper ions in wastewater using calcined waste eggshells as natural adsorbents
Source: Sci Rep. 2023 Jan 9;13:437. doi: 10.1038/s41598-023-27682-5 (PMC9829870; doi:10.1038/s41598-023-27682-5)
Supplement: Supplementary file 1 — Supplementary Information. [file 41598_2023_27682_MOESM1_ESM.docx]

Annex

Table A1. Second-order model equations for the response surfaces fitted to the data points as the adsorption rate of four different eggshells in copper ion solution and the analysis of regression

Table A2. Analysis of variance for effect factors related to the adsorption of eggshell in copper ion solution

Table A3. Analysis of variance for effect factors related to the adsorption of eggshell membrane in a copper ion solution

Table A4. Analysis of variance for effect factors related to the adsorption of eggshell with a membrane (ESWM) in a copper ion solution.

Table A5. Analysis of variance for effect factors related to the adsorption of calcined eggshell (CSE4) in a copper ion solution

Figure A1. Effect of four eggshells on the adsorption rate under RSM optimum conditions (metal concentration, reaction time, and adsorbent dose).

| Table A1. Second-order model equations for the response surfaces fitted to the data point as the adsorption rate of four different eggshells in a copper ion solution. | | | | |
| --- | --- | --- | --- | --- |
| Regression coefficient for adsorption | | | | |
|  | Eggshell (ES) | Eggshell membrane (ESM) | Eggshell with a membrane (ESWM) | Calcined eggshell  (CES4) |
| Cofficirnt | Estimates | | | |
| Constant | -53.625 | 72.8875 | 146.225 | 105.149 |
| A: Reaction time | 0.71007 | 0.38264 | -5.60764 | -0.317986 |
| B: Concentration | -0.20167 | -1.47333 | 1.97783 | 0.0289722 |
| C: Adsorption dose | 154.525 | -0.085 | -162.275 | -0.244033 |
| AA | -0.0616 | 0.00622 | 0.0917 | 0.00445833 |
| AB | 0.0005 | -0.00975 | 0.0025 | -0.0001 |
| AC | 1.3125 | -0.00163 | 0.44167 | -0.00180556 |
| BB | 0.00729 | 0.00567 | -0.01179 | -0.00040667 |
| BC | 0.072 | 0.01042 | 0.054 | 0.00627778 |
| CC | -65.0833 | -0.00126 | 52.5167 | -0.0177984 |
|  |  |  |  |  |

Table A2. Analysis of variance for effect factors related to the adsorption of eggshell (ES) in a copper ion solution

| Source | Sum of squares | Degrees of freedom | Mean square | F-Ratio | *p*-Value |
| --- | --- | --- | --- | --- | --- |
| A: Reaction time | 73.81120 | 1 | 73.81120 | 0.29 | 0.6130 |
| B: Concentration | 2093.05000 | 1 | 2093.05000 | 8.24 | 0.0350* |
| C: Adsorption dose | 63.28130 | 1 | 63.28130 | 0.25 | 0.6389 |
| AA | 290.55400 | 1 | 290.55400 | 1.14 | 0.3338 |
| AB | 0.00900 | 1 | 0.09000 | 0.00 | 0.9857 |
| AC | 248.06300 | 1 | 248.06300 | 0.98 | 0.3685 |
| BB | 76.58010 | 1 | 76.58010 | 0.30 | 0.6066 |
| BC | 3.24000 | 1 | 3.24000 | 0.01 | 0.9145 |
| CC | 977.50200 | 1 | 977.50200 | 3.85 | 0.1071 |
| Total error | 1270.38000 | 5 | 254.07700 |  |  |
| Total (corr.) | 5095.58000 | 14 |  |  |  |

*Significant at 5% level

R-squared = 75.0689 %

R-squared (adjusted for d.f.) = 30.1929%

Standard Error of Est. = 15.9398

Mean absolute error = 8.25889

Durbin- Watson statistic = 1.91539 (P = 0.6481)

Lag 1 residual autocorrelation = -0.039536

Table A3. Analysis of variance for effect factors related to the adsorption of eggshell membrane (ESM) in a copper ion solution

| Source | Sum of squares | Degrees of freedom | Mean square | F-Ratio | *p*-Value |
| --- | --- | --- | --- | --- | --- |
| A: Reaction time | 2.88000 | 1 | 2.88000 | 0.04 | 0.8452 |
| B: Concentration | 894.64500 | 1 | 894.64500 | 13.14 | 0.0151* |
| C: Adsorption dose | 6.84500 | 1 | 6.84500 | 0.10 | 0.7640 |
| AA | 2.96314 | 1 | 2.96314 | 0.04 | 0.8430 |
| AB | 34.22250 | 1 | 34.22250 | 0.50 | 0.5100 |
| AC | 3.80250 | 1 | 3.80250 | 0.06 | 0.8226 |
| BB | 46.42310 | 1 | 46.42310 | 0.68 | 0.4466 |
| BC | 678.60200 | 1 | 678.60200 | 9.97 | 0.0252* |
| CC | 36.73390 | 1 | 36.73390 | 0.54 | 0.4956 |
| Total error | 1270.38000 | 5 | 68.09430 |  |  |
| Total (corr.) | 5095.58000 | 14 |  |  |  |

* Significant at 5% level

R-squared = 83.4266%

R-squared (adjusted for d.f.) = 53.5944 %

Standard Error of Est. = 8.25193

Mean absolute error = 3.84444

Durbin- Watson statistic = 2.07044 (P = 0.1183)

Lag 1 residual autocorrelation = -0.0957534

Table A4. Analysis of variance for effect factors related to the adsorption of eggshell with a membrane (ESWM) in a copper ion solution.

| Source | Sum of squares | Degrees of freedom | Mean square | F-Ratio | *p*-Value |
| --- | --- | --- | --- | --- | --- |
| A: Reaction time | 202.00500 | 1 | 202.00500 | 4.59 | 0.0850 |
| B: Concentration | 4413.30000 | 1 | 4413.3000 | 100.36 | 0.0002** |
| C: Adsorption dose | 147.06100 | 1 | 147.06100 | 3.34 | 0.1270 |
| AA | 643.75400 | 1 | 643.75400 | 14.64 | 0.0123* |
| AB | 2.25000 | 1 | 2.25000 | 0.05 | 0.8300 |
| AC | 28.09000 | 1 | 28.09000 | 0.64 | 0.4604 |
| BB | 200.60000 | 1 | 200.60000 | 4.56 | 0.0858 |
| BC | 1.82250 | 1 | 1.82250 | 0.04 | 0.8467 |
| CC | 636.46200 | 1 | 636.46200 | 14.47 | 0.0126* |
| Total error | 219.88400 | 5 | 43.9768 |  |  |
| Total (corr.) | 6522.74000 | 14 |  |  |  |

* Significant at 5% level

** Significant at 1% level

R-squared = 96.629%

R-squared (adjusted for d.f.) = 90.5611%

Standard Error of Est. = 6.6315

Mean absolute error = 3.35889

Durbin- Watson statistic = 2.74314 (P = 0.7929)

Lag 1 residual autocorrelation = -0.468132

Table A5. Analysis of variance for effect factors related to the adsorption of calcined eggshell (CSE4) in a copper ion solution

| Source squares | Sum of squares | Degrees of freedom | Mean | F-Ratio | *p*-Value |
| --- | --- | --- | --- | --- | --- |
| A: Reaction time | 0.10125 | 1 | 0.10125 | 0.02 | 0.9010 |
| B: Concentration | 23.4613 | 1 | 23.4613 | 3.96 | 0.1031 |
| C: Adsorption dose | 47.045 | 1 | 47.045 | 7.95 | 0.0371* |
| AA | 11.7426 | 1 | 11.7426 | 1.98 | 0.2180 |
| AB | 0.04 | 1 | 0.04 | 0.01 | 0.9377 |
| AC | 0.4225 | 1 | 0.4225 | 0.07 | 0.8000 |
| BB | 3.81641 | 1 | 3.81641 | 0.64 | 0.4584 |
| BC | 31.9225 | 1 | 31.9225 | 5.39 | 0.0678 |
| CC | 7.6741 | 1 | 7.6741 | 1.30 | 0.3064 |
| Total error | 29.5892 | 5 | 5.91783 |  |  |
| Total (corr.) | 1746.62000 | 14 |  | | |

* Significant at 5% level

R-squared = 81.2286%

R-squared (adjusted for d.f.) = 47.4402%

Standard Error of Est. = 2.43266

Mean absolute error = 1.22556

Durbin- Watson statistic = 1.29822 (P = 0.0921)

Lag 1 residual autocorrelation = 0.317934


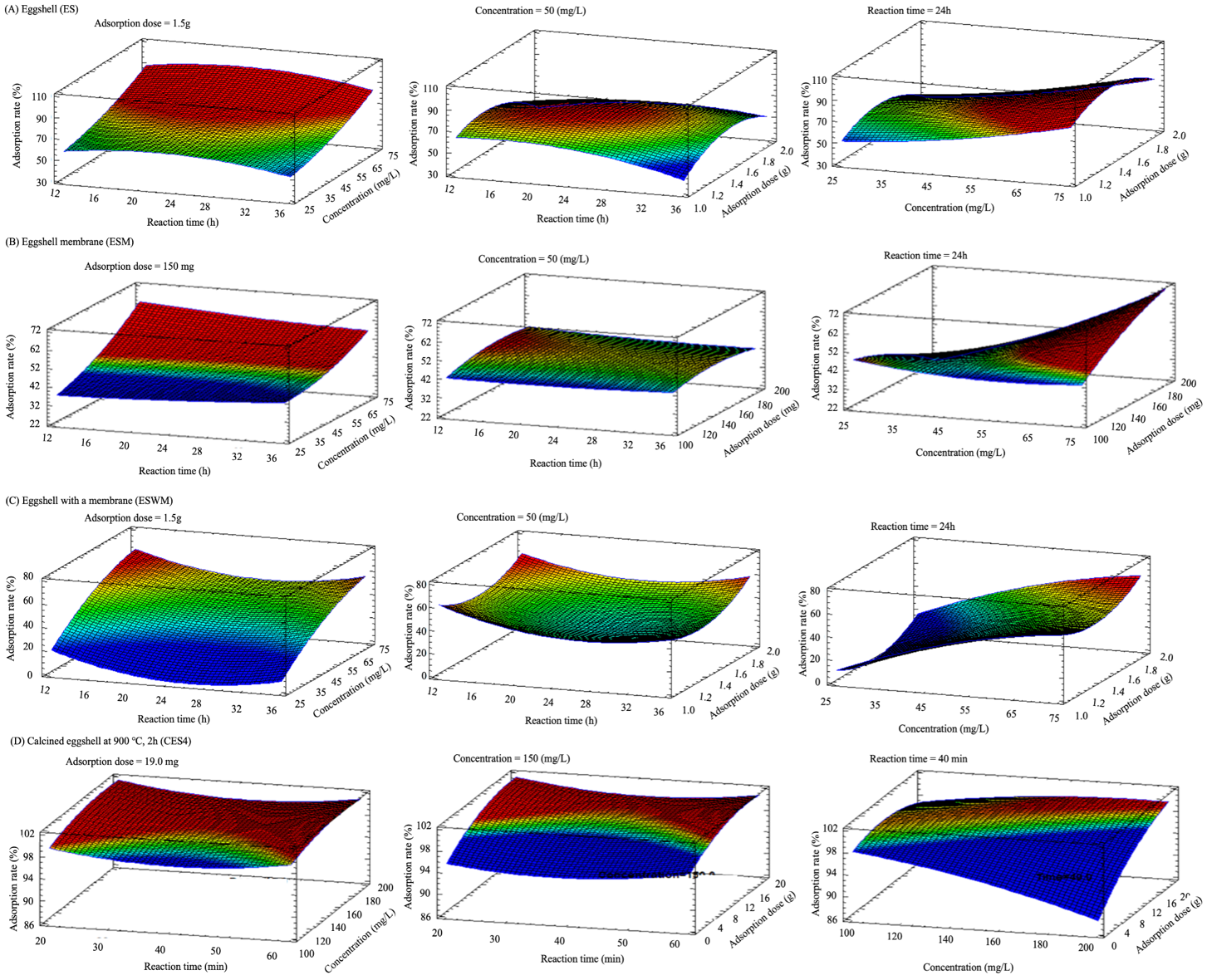


Figure A1. Effect of four eggshells on the adsorption rate under RSM optimum conditions (metal concentration, reaction time, and adsorbent dose).
